# Application of CAR-T Cell Therapy beyond Oncology: Autoimmune Diseases and Viral Infections

**DOI:** 10.3390/biomedicines9010059

**Published:** 2021-01-09

**Authors:** Ekaterina Zmievskaya, Aygul Valiullina, Irina Ganeeva, Alexey Petukhov, Albert Rizvanov, Emil Bulatov

**Affiliations:** 1 Institute of Fundamental Medicine and Biology, Kazan Federal University, 420008 Kazan, Russia; ekazmievskaya@gmail.com (E.Z.); aigul1692@mail.ru (A.V.); kovirka1995@gmail.com (I.G.); rizvanov@gmail.com (A.R.); 2 Almazov National Medical Research Center, Institute of Hematology, 197341 Saint Petersburg, Russia; alexeysakhalin@gmail.com; 3 Shemyakin-Ovchinnikov Institute of Bioorganic Chemistry, Russian Academy of Sciences, 117997 Moscow, Russia

**Keywords:** chimeric antigen receptor, T lymphocytes, CAR-T, immunotherapy, autoimmune diseases, human immunodeficiency virus, SARS-CoV-2

## Abstract

Adoptive cell transfer (ACT) has long been at the forefront of the battle with cancer that began last century with the therapeutic application of tumor-infiltrating lymphocytes (TILs) against melanoma. The development of novel ACT approaches led researchers and clinicians to highly efficient technologies based on genetically engineered T lymphocytes, with chimeric antigen receptor (CAR)-T cells as the most prominent example. CARs consist of an extracellular domain that represents the single-chain variable fragment (scFv) of a monoclonal antibody (mAb) responsible for target recognition and the intracellular domain, which was built from up to several signaling motifs that mediated T cell activation. The number of potential targets amenable for CAR-T cell therapy is expanding rapidly, which means that the tremendous success of this approach in oncology could be further translated to treating other diseases. In this review, we outlined modern trends and recent developments in CAR-T cell therapy from an unusual point of view by focusing on diseases beyond cancer, such as autoimmune disorders and viral infections, including SARS-CoV-2.

## 1. Introduction

Adoptive cell transfer (ACT) has a rich history in the development of cancer treatments. Last century, an immune therapy approach against melanoma was developed based on enhancement of tumor-infiltrating lymphocytes (TILs). Nowadays, the development of ACTs led researchers and clinicians to technologies based on genetically engineered T lymphocytes [1]. One of the most successful examples of ACT are chimeric antigen receptor (CAR)-T cells that fight against CD19 B cell antigens. CAR-T cells have been approved by the U.S. Food and Drug Administration (FDA) as a treatment of refractory pre-B cell acute lymphoblastic leukemia to diffuse large B cell lymphoma [2].

CARs are synthetic constructs that consist of extracellular domains for target cell recognition represented by a single-chain variable fragment (scFv) from a monoclonal antibody (mAb). The intracellular part includes up to several signaling motifs capable of T cell activation [3]. One of the main advantages of CAR-T cells is major histocompatibility complex (MHC)-independent antigen recognition. The multi-step process to manufacture CAR-T cells for therapeutic application begins with separating T cells from a patient’s peripheral blood, followed by a viral or non-viral insertion of CAR genes into the T cell genome. Next, the CAR-T cells expand and the cell product is infused back into the patient [1].

The number of available targets for CAR-T cell therapy is expanding rapidly [4]. Major successes in oncology have led to a growing scientific and clinical interest in using CAR-Ts as a treatment for other types of diseases. In this paper, we attempted to overview the modern approaches of CAR-T cell therapy from an unusual point of view by focusing on diseases beyond cancer. Despite differences in etiology, there are common features in pathogenesis of the diseases described below that potentially allow them to be treated with CAR-T cells. This includes a specified disease-linked cellular component that marks cells as infected, hyperactive, or overexpanded. Moreover, in many instances, CAR-Ts operate as a powerful alternative to human immune system that are partly dysfunctional due to a disease. 

## 2. Chimeric Antigen Receptor (CAR)-T Cells and Autoimmunity

**Figure 1:**
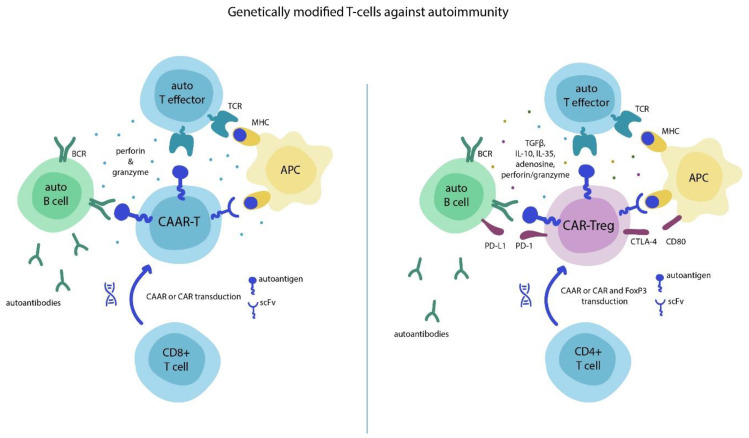
Mechanisms of chimeric autoantibody receptor (CAAR)-T and chimeric antigen receptor (CAR)-Treg activity. CAAR-Ts recognize autoreactive B and T cells with receptors to autoantigens, as well as autoreactive antigen-presenting cells (APCs), through an major histocompatibility complex (MHC):autoantigen complex. They then execute the cytotoxic perforin/granzyme-based immune attack. CAR-Tregs identify targets in the same manner, but their action can be considered as immunosuppressive rather than cytotoxic because it is associated with cytokines and immune checkpoint proteins. *Abbreviations: APC—antigen-presenting cell; BCR—B cell receptor; CAAR—chimeric autoantibody receptor; CTLA-4—cytotoxic T-lymphocyte antigen 4; MHC—major histocompatibility complex; TCR—T cell receptor.*

Pathogenesis of many autoimmune diseases (AIDs) has still not been determined precisely, but there is no doubt that T cell tolerance failure plays a central role in this process [5]. The mechanisms underlying the loss of immunological self-tolerance in AIDs include activation of autoreactive B cell clones that produce autoantibodies promoting tissue damage, and suppression of cytotoxic or regulatory T cells. These mechanistic aspects of disease pathogenesis are reflected in the concept of therapeutic application of anti-autoimmune CAR-T cells aimed at eliminating autoreactive clones of immune cells (Figure 1).

The chimeric autoantibody receptor (CAAR), also known as the B cell antibody-targeting receptor (BAR), represents a variation of modified CAR. Unlike the scFv domain, CAAR functions as a target for autoreactive B cells and defines the selective cytotoxicity of CAAR-T cells only against immune cells that carry receptors to specific autoantigen without inducing immunosuppression [6,7]. Such a strategy is supposed to lead to the direct elimination of surface immunoglobulin memory B cells, and indirect elimination of short-lived plasma cells that produce the disease-causing autoantibodies. A similar immune cell eradication strategy can be applied by inserting the CAAR motif into the recognition mAb-specific domain for the MHC:peptide complex of autoreactive antigen-presenting cells (APCs) or T cell receptors (TCR) for CD8+ T cells [8,9].

Another interesting concept for the restoration of immune tolerance implies “switching” T cell phenotype from cytotoxic to regulatory, since Tregs are usually suppressed in AIDs. CAR-Tregs represent CAR-T cells converted to Tregs by transduction of FOXP3 (along with CAR), which is a member of the FOX protein family that controls pathways responsible for the development and function of regulatory T cells [10]. CAR-Tregs recognize and regulate autoimmune T lymphocytes through induction of anergy, immunological ignorance, and clonal deletion.

These approaches were tested in a number of preclinical studies, e.g., Ellebrecht et al. tried to use CAAR-T cells to treat AIDs in case of pemphigus vulgaris (PV), an autoimmune disease that causes painful blistering on the skin and mucous membranes [6]. PV is a life-threatening autoimmune disease caused by autoantibodies to keratinocyte adhesion protein Dsg3. Anti-Dsg3 B cell receptors (BCRs) are expressed on pathogenic memory B cells, found in PV. CAAR-T cells with Dsg3 autoantigen as the extracellular domain were produced and showed high efficiency in vitro in antibody-secreting hybridomas, as well as in vivo in PV mouse models. Specific elimination of anti-Dsg3 B cells, even in the presence of circulating blocking antibodies, was observed without any toxic off-target activity. A clinical trial seeking to determine the maximum tolerated dose of Dsg3-CAAR-T in mucosal-dominant PV patients is presently recruiting for Phase I (NCT04422912) (Table 1).

A similar concept was proposed by Parvathaneni et al. for the treatment of Hemophilia A (HA) [7]. Recombinant or plasma-derived factor VIII (FVIII) replacement is a standard therapy for this disorder; however, up to 30% of patients are immunologically intolerant to human FVIII and produce neutralizing anti-FVIII antibodies. FVIII-expressing BAR CD8+ T cells were examined in B cell hybridomas and mouse models of HA and were shown to prevent the generation of anti-FVIII antibodies in vivo and retain FVIII activity in the presence of blocking FVIII antibodies in vitro.

Many studies related to CAR-T therapy against AIDs are focused on Type 1 diabetes (T1D), a T cell-mediated autoimmune disease in which both CD4+ and CD8+ T cells are involved in the destruction of insulin-producing islet β cells. Fishman et al. proposed the design of a peptide/β2m/CD3-ζ receptor that targets CD8+ T cell clones carrying TCRs specific to the peptide of interest, e.g., fragments of insulin-B chain InsB15–23, or islet-specific glucose-6-phosphatase catalytic subunit-related protein IGRP206–214 [8]. The engineered CD8+ T lymphocytes were able to destroy insulin-reactive or IGRP-reactive T cells in vitro. Moreover, InsB15–23/β2m/CD3ζ T cells demonstrated a significant reduction in the frequency of diabetes incidence in non-obese diabetic (NOD) mice in vivo. Zhang et al. presented a similar strategy aimed at APCs involved in T1D [9]. They replaced CAR extracellular domain with mAb287 specific to InsB9-23 and I-Ag7, a fragment of MHCII in NOD mice. The 287 CAR-T cells displayed cytotoxic activity against APCs in vitro and then induced a delay of onset T1D in NOD mice. Tenspolde et al. suggested another approach for remission and treatment of T1D based on CAR-Tregs generated from anti-insulin CD4+ CAR-Ts by transduction of FOXP3 [10]. Insulin-targeted CAR-Tregs migrated to the pancreas and acted as regulators at the local site of cell destruction. However, the strong immunosuppressive capacity of CAR-Tregs was found only in vitro but not in vivo, despite a prolonged persistence in diabetic mice.

The abovementioned concept was also examined as a potential treatment for multiple sclerosis (MS) since one of the central roles in its pathogenesis is autoreactive T cells recognizing myelin epitopes. Fransson et al. designed CAR-Tregs to target myelin oligodendrocyte glycoprotein (MOG) in which the role of the CAR-αMOG receptor was to bring Tregs in close proximity with MOG+ oligodendrocytes to prevent immune attacks against them [11]. CAR-Tregs were able to infiltrate the central nervous system (CNS) in a mouse model and reduce the active inflammation process, thereby eliminating symptoms and potentially suppressing effector T cells, as shown by co-culturing experiments.

CAR-Tregs were also reported to be effective for the treatment of ulcerative colitis associated with overexpression of carcinoembryonic antigen (CEA), a well-known tumor marker of colon inflammation [12]. Blat et al. explored CEA as a target for CAR-Treg redirection and demonstrated sufficient immunosuppressive functions of anti-CEA CAR-Tregs in vitro and protected mice from death in a dose-dependent manner.

Importantly, three ongoing clinical trials are focused on the application of CAR-T cell therapy against autoimmune diseases by depleting the whole B cell population instead of the point autoreactive clone elimination. NCT04146051 evaluates autologous Descartes-08 CAR-T cells that target B cell maturation antigens (BCMA) for the treatment of generalized myasthenia gravis, which is a chronic, autoimmune, and neuromuscular condition that causes muscle weakness in different parts of the body. NCT03030976 is based on classical anti-CD19 CAR-Ts to deplete B cells in CD19+ systemic lupus erythematosus (SLE), in which the immune system attacks its own tissues, causing widespread inflammation and tissue damage in the affected organs. Another clinical study (NCT03605238) used tandem anti-CD19 and anti-CD20 CAR-Ts to treat neuromyelitis optica spectrum disorder (NMOSD), which is a chronic disorder of the brain and spinal cord dominated by inflammation of the optic nerve (optic neuritis) and inflammation of the spinal cord (myelitis). In addition, the NCT04561557 study is based on anti-BCMA CAR-T cells for elimination of plasma cells in patients with AQP4-IgG-seropositive NMOSD who suffer recurrent attacks from conventional treatments.

## 3. Allergy and Asthma

Allergic diseases (ADs) and asthma are characterized by the domination of the Th2 immune response, which can be modulated by Tregs [13]. IgE produced by B cells play an important role in the pathogenesis of ADs and may be targeted by CAR-Ts.

The main event in the initiation of allergic reactions is the binding of IgE to its respective IgE receptor FcεRI, which are expressed on mast cells, eosinophils, and basophils that cause degranulation and release of inflammatory mediators resulting in type I hypersensitivity reactions and allergic symptoms [14]. Ward et al. proposed that IgE-expressing cells (germinal center B cells, plasmablasts, plasma cells, and memory B cells) can be targeted by CAR-Ts through the recognition of the transmembrane form of IgE (mIgE). For this purpose, CARs that include the extracellular domain of FcεRI α chain (FcεRIα) for mIgE binding were designed and examined in vitro. The FcεRIα-based low affinity CD8+ CAR-Ts were shown to be capable of mediating potent primary T cell responses against mIgE+ target cells.

Pathogenesis of allergic asthma is associated with a low number of Tregs and reduced immunosuppressive activity, as well as excessive Th2 cell-dominated responses to allergens leading to airway inflammation, hyper-reactivity, and reversible obstruction [15]. Skuljec et al. attempted to redirect Tregs to lungs and initiate their activation by CAR that recognizes CEA, a glycoprotein presented on the surface of adeno epithelia in lungs and the gastrointestinal tract. Anti-CEA CAR-Tregs were tested in vitro and on a CEA-transgenic mouse model of asthma. The results suggested increased suppression of effector CAR-Ts and reduced expression of Th2 cytokine, as well as allergen-specific IgE, airway hyper-reactivity, eosinophilic airway inflammation, and enhanced mucus production.

## 4. Infectious Diseases

The main function of CD8+ T cells is their ability to eliminate foreign cells and agents, making them attractive CAR-Ts for treating infectious diseases [16]. Patients with chronic hepatitis B virus (HBV) normally do not develop sufficiently strong immune responses, which can lead to the development of liver cancer [17]. Krebs et al. designed and tested CAR-Ts with an S domain receptor (S-CARs) for all 3 HBV envelope proteins (S, M, and L), which, together, formed an HBV surface antigen (HBsAg) that was exposed on the surface of the infected cells. After the infusion of S-CARs in mice, the authors observed a decrease in the number of hepatocytes with cytoplasmic expression of HBV core protein, virions circulating in the bloodstream, and replicative forms of HBV DNA. However, soon after the treatment, S-CAR-Ts were completely exhausted and viral activity increased again due to the immune response against the human S-CAR domains. To address this issue, Festag et al. used special immunocompetent mice tolerant to allogeneic CAR domains to demonstrate the sustained antiviral effect [18]. In another study, Kruse et al. designed a CAR specific to HBsAg and then evaluated its ability to recognize HBV+ cells and HBsAg particles in vitro, examining the efficacy against HBV-infected hepatocytes in human liver chimeric mouse model [19]. The anti-HBs-G4m CAR-T cells were shown to recognize HBsAg particles and HBV+ cells in vitro, and efficiently reduce HBV-DNA and HBsAg levels in vivo.

Chronic hepatitis C virus (HCV) infection is a medical indication for liver transplantation for end-stage chronically infected patients who are nonresponsive to current therapies. For many HCV cases, there remains a high risk of post-treatment reinfection, which implies a need for the development of alternative therapeutic approaches. Sautto et al. developed CAR-T cells that recognize HCV E2 glycoprotein (HCV/E2), a major target of the host immune response and one of the most variable viral proteins exposed on the surface of infected cells [20]. These anti-HCV/E2 CAR-T cells demonstrated substantial cytotoxic activity against HCV infected cells.

Infection by human cytomegalovirus (HCMV) and its reactivation is a major cause of mortality after hematopoietic stem cell and solid organ transplantation. Proff et al. attempted to redirect T cells to HCMV-glycoprotein B (gB); however, despite the activation, anti-gB CAR-T cells were not able to lyse infected cells in vitro [21]. Anti-gB CAR-T cells were found to mediate inhibition of HCMV replication, independent of their cytotoxic effector functions via the combined action of IFNγ and TNFα released by the activated CAR-Ts. Patients after an organ transplantation tend to experience the same problem caused by opportunistic invasive fungal infections (IFIs), e.g., aspergillosis. Kumaresan et al. modified the CAR design with Dectin-1 extracellular domain (D-CAR) to achieve recognition of carbohydrates expressed on the fungi surface [22]. Based on the results of in vitro and in vivo experiments, they concluded that D-CAR-T cells can directly target and treat aspergillus infections.

The Influenza A virus, which gave rise to swine flu and avian flu, was studied by Talbot et al. as a potential therapeutic application for CAR-T cells [23]. In this study, a conserved region of M2e protein expressed on the surface of the infected cells was selected as a target in both in vitro and in vivo experiments, resulting in a reduction of the viral titer in murine lungs.

## 5. HIV

Human immunodeficiency virus type 1 (HIV-1) replication can be successfully suppressed via combination antiretroviral therapy (cART); however, complete elimination of the latent reservoir of infected cells still remains a major problem. CD8+ T cellular cytotoxic activity against HIV-1 infected cells plays a critical role in managing HIV-1 infection; therefore, attempts to apply CAR-Ts for HIV-1 treatment provide a promising new therapeutic avenue that catches up with its use in oncology [24,25].

**Figure 2:**
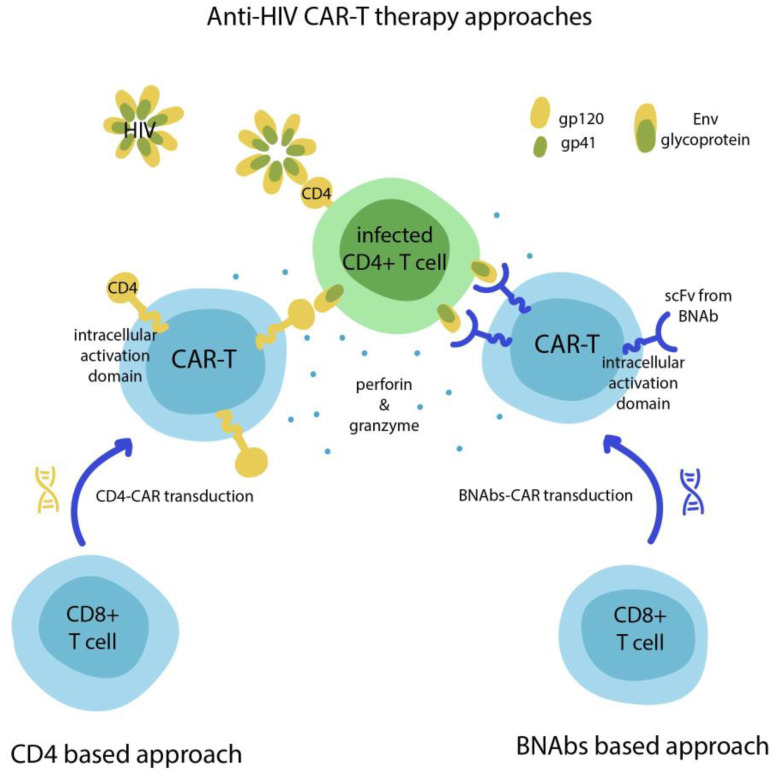
Two main approaches of anti-human immunodeficiency virus CAR-T therapy. The first one is based on gp120-CD4 interaction (**left**), and the second uses single-chain variable fragments from broadly neutralizing antibodies (BNAbs) targeting gp120 and gp41 regions of Env protein (**right**).

The main target for anti-HIV-1 CAR-T therapy is considered to be the gp120 region of the HIV Envelope (Env) glycoprotein expressed on the surface of HIV infected cells (Figure 2). Deeks et al. designed an HIV-targeting receptor that consisted of the transmembrane and extracellular domains of human CD4 (which targets gp120) [26]. In vitro studies showed that CD4+ CAR-T cells were able to suppress the viral replication in HIV-infected T cells and macrophages, as well as destroy HIV-infected T cells. Clinical trials with undetectable plasma viremia demonstrated a low viral rebound in patients receiving gene-modified cell therapy, as well as a significant decrease in gut HIV DNA, but not mRNA, suggesting that CD4+ CAR-Ts can indeed impact tissue viral reservoirs.

Since the early trials began, the structure and function of clinically tested CARs have vastly improved. Sahu et al. developed a second generation CD4+ CAR-Ts that, in vitro, eliminated not only infected and HIV-producing cells but the entire latent cell population [27]. Leibman et al. revealed that the components of novel CARs such as expression vector, promoter, transmembrane, and costimulatory domains enable superior control over HIV infection in vitro [28]. Treatment of mice with optimized CAR-T cells lead to significantly higher CD4+ CAR-T cell numbers and enhanced CD8+ CAR-T cell proliferation after HIV infection, and was associated with almost 90% less HIV RNA compared to clinical trials of CD4ζ CAR.

Despite the overall limited in vivo efficacy, the interest towards the application of anti-HIV CAR-T has significantly intensified over the past few years due to the outstanding success of CAR-T therapy in cancer treatment. Anti-HIV CARs based on a single-chain fragment of the human anti-gp120 mAb were used by Masiero et al., who designed and tested such constructs in vitro and achieved promising results [24]. An interesting example of the new generation of anti-HIV CARs reported by Ali et al. is based on HIV-1 broadly neutralizing antibodies (BNAbs) that target gp120 and gp41 motifs [29]. These BNAb-based CAR-T cells exhibited substantial antiviral activity in vitro through recognition and destruction of HIV-1-infected target cells. In another study, Liu et al. demonstrated the capacity of BNAbs-based CAR-Ts to suppress HIV-1 viral rebound after withdrawal of the antiviral treatment [30]. For that, they used an in vitro model that validated the elimination of autologous reactivated HIV-1-infected CD4+ T cells isolated from patients receiving suppressive cART. In addition to this, Hale et al. developed a novel in vitro approach for homology-directed recombination of the anti-HIV-CAR gene expression cassette that disrupts the CCR5 locus and protects the engineered cells from HIV infection [31].

Several research groups attempted to combine CD4-based and BNAbs-based CARs into one construct to allow dual specificity and produce highly potent anti-HIV CAR-T cells. Liu et al. reported bispecific CD4+ CAR-Ts consisting of human CD4 (binds native Env on virions) attached to an scFv of the human anti-gp120 mAb (binds and neutralizes infected cells) [32]. This approach displayed strong virus suppression, low immunogenicity, and the lack of receptor activity associated with HIV entry. Further, Ghanem et al. achieved improved potency compared to bispecific CARs by replacing scFv with carbohydrate recognition domain (CRD) of a human C-type lectin, which bound to the dense oligomannose patch commonly presented on Env [33].

Currently, two Phase I clinical trials are recruiting to evaluate the safety and efficacy of CAR-T cell therapy for HIV treatment. The NCT03240328 trial enrolls HIV patients whose plasma HIV has been successfully suppressed after cART and is expected to enhance the reconstitution of HIV-specific immune function to assist the eradication of HIV reservoirs. The NCT03980691 trial evaluates the combined effect of chidamide with CAR-T or TCR-T cell therapy on HIV-1 latent reservoirs. The NCT04648046 trial that will evaluate the efficacy of bispecific anti-gp120 CAR-T cells has not yet started.

## 6. COVID-19

The approach of using CAR-transduced immune cells against virus infected cells has recently attracted the attention of the scientific community for the treatment of COVID-19, a contagious disease caused by severe acute respiratory syndrome coronavirus 2 (SARS-CoV-2). A promising therapeutic approach is based on natural killer (NK) cells, which are effector lymphocytes of the innate immunity with the main function to destroy tumor and virally infected cells. NK cells function through either direct recognition of viral proteins or inhibitory NK receptor signaling in the case of downregulated MHC class I on the infected cell surface [34].

Ma et al. tried to develop CAR-NK cells to target the SARS-CoV-2 spike protein with the CR3022 scFv domain, which is a strong neutralizing antibody for SARS-CoV-1 and SARS-CoV-2 [35]. They found that CR3022-CAR-NK cells were able to eliminate SARS-CoV-2 infected cells in vitro.

**Figure 3:**
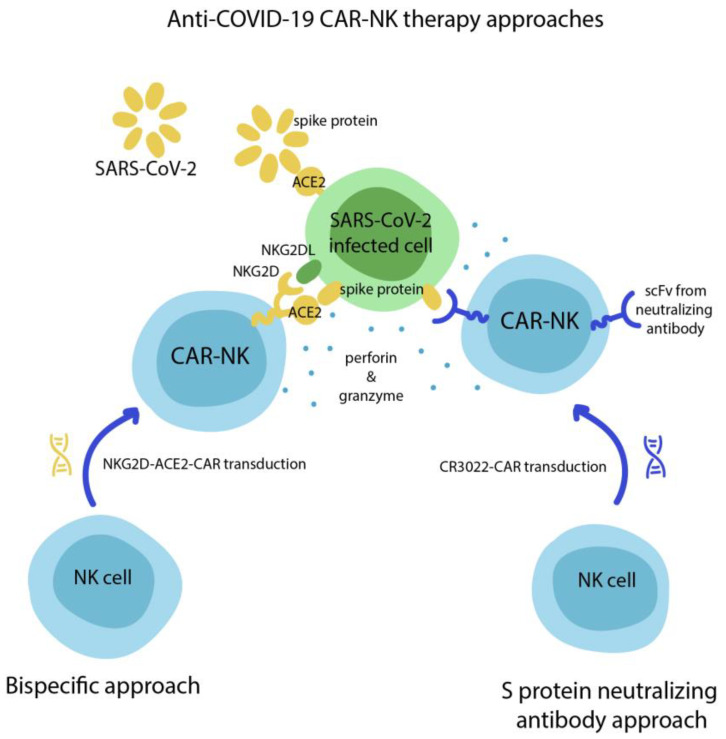
Experimental approaches for anti-COVID-19 CAR-natural killer (NK) therapy. One is based on bispecific recognition of infected cells through ACE2-S protein and NKG2D-NKG2DL interactions (**left**), while another uses classical single-chain variable fragments from an S protein neutralizing antibody (**right**).

Currently, there is only one clinical trial (NCT04324996) evaluating bispecific NKG2D-ACE2 CAR-NK cells as a promising COVID-19 therapy. NKG2D is the universal activating receptor of NK cells that recognize the infected cells, while ACE2 is the receptor that binds the SARS-CoV-2 spike protein (Figure 3).

In addition to the expanding scope of clinical applications, lessons learned from CAR-T therapy helped us tackle the complications in other diseases. An interesting example is the cytokine release syndrome (CRS) caused by hypersecretion of proinflammatory cytokines (e.g., IL-6 or IFNγ) by CAR-T cells activated in response to tumor recognition [36]. CRS is a severe side effect of CAR-T cell therapy accompanied by symptoms such as fever, malaise, myalgias, hypoxia, hypotension, and renal impairment. Interestingly, COVID-19 is associated with a similar complication called cytokine storm syndrome (CSS), characterized by hypercytokinemia (predominantly IL-6) and immune dysregulation that leads to multiple organ failure and high mortality rates. The significant similarity of these two syndromes allows for therapy substitution, e.g., Tocilizumab, an IL-6-receptor antagonist approved by the FDA for CRS treatment, can also be successfully used for CSS in COVID-19 [37].

## 7. Cardiac Fibrosis

Fibrosis is a pathological process associated with the hyperactivation and expansion of fibroblasts, as well as the deposition of extracellular matrix components. Many pathological stimuli (e.g., ischemic damage or hemodynamic stress) can lead to cardiac fibrosis and the progression of heart failure. Currently, there is still no available targeted therapy to treat cardiac fibrosis. Aghajanian et al. assessed the potency of CAR-T cells that target activated heart fibroblasts through the recognition of fibroblast activation protein (FAP) in mice with induced hypertensive cardiac injury and fibrosis [38,39]. They found that anti-FAP CAR-Ts induce significant reductions or even the complete elimination of cardiac fibrosis. They also can induce a partial rescue of both systolic and diastolic cardiac functions with no adverse effects.

## 8. Conclusions

The ever-growing progress in our understanding of various pathologies and their fundamental molecular mechanisms pushes the application boundaries of cellular immunotherapy far beyond oncology. A plethora of molecules confirmed as therapeutic targets for a wide range of diseases renders immune cell therapy as an attractive and highly promising treatment option (Table 2). The rapid development of genetic engineering technologies now allows the researchers to test their ambitious concepts by implementing novel and often revolutionary clinical approaches towards previously untreatable diseases.

The application of CAR-T cell technology is eagerly awaited in numerous fields, e.g., for the treatment of viral infections in patients with primary immune deficiency (PID) [40]. Currently, PID immunotherapy includes selection and expansion of antigen-specific T cells for the treatment of HCMV, mononucleosis (Epstein-Barr virus disease), and adenoviral infections of respiratory and intestinal tracts. In many of these cases the autologous cell therapy is the only option due to the risks of graft-versus-host disease (GvHD) related to non-autologous transfusions.

In addition, investigation of CAR-T cell action in immune dysfunction caused by autoimmune diseases or HIV can help understand mechanisms of tumor immune evasion. For example, exploring migration of CAR-T cells into the tumor stroma and the lymph node HIV sanctuaries will generate mutually complementary new knowledge about the molecular mechanisms involved in both pathogenic conditions [41].

Despite a large number of common issues associated with CAR-T cell therapy that still remain largely unresolved (e.g., difficulty and expensiveness of manufacturing), the overall great promise of this approach is supported by encouraging clinical results and solid technological basis [42]. The ongoing research in this field offers hope for many yet incurable diseases that could potentially be eradicated with novel CAR-T cell therapies, and paves prospective avenues for future breakthroughs in their clinical applications.

## Figures and Tables

**Table 1:** The list of clinical trials of chimeric antigen receptor (CAR)-T cell therapy beyond oncology.

Disease	Target/Approach	NCT, Current Status
Mucosal-Dominant Pemphigus Vulgaris	Clone-specific anti-Dsg3 CAAR-T	NCT04422912, Phase I recruiting
Generalized Myasthenia Gravis	Non-specific anti-BCMA CAR-T	NCT04146051, Phase I, II recruiting
Systemic Lupus Erythematosus	Non-specific anti-CD19 CAR-T	NCT03030976, Phase I, unknown
Neuromyelitis Optica Spectrum Disorder	Non-specific tandem anti-CD19 and anti-CD20 CAR-T	NCT03605238, Phase I, withdrawn
	Non-specific anti-BCMA CAR-T	NCT04561557, Phase I recruiting
Human Immunodeficiency Virus	Anti-gp120 BNAbs based CAR-T	NCT03240328, Phase I recruiting
		NCT03980691, Phase I recruiting
	Anti-gp120 dual CAR-T	NCT04648046, Phase I not yet recruiting
COVID-19	Bispecific anti-ACE2 and anti-NKG2D CAR-NK	NCT04324996, Phase I, II recruiting

**Table 2:** The list of diseases that can potentially be treated with CAR-T cell therapy and the respective target molecules.

Disease	Target for CAR-T Cell
**Autoimmunity and allergy**	
Pemphigus vulgaris	Keratinocyte adhesion protein Dsg3 [6]
Hemophilia A	Anti-FVIII antibody [7]
Type 1 diabetes	Insulin-B chain [8,9,10] Islet-specific glucose-6-phosphatase catalytic subunit-related protein [8]
Multiple sclerosis	Myelin oligodendrocyte glycoprotein [11]
Ulcerative colitis	Carcinoembryonic antigen [12]
Allergy	Transmembrane form of IgE [14]
Allergic asthma	Carcinoembryonic antigen [15]
**Infectious diseases**	
HBV	S domain of HBsAg [17,18,19]
HCV	Glycoprotein E2 [20]
HCMV	Glycoprotein B [21]
Aspergillus	Carbohydrate of cell surface [22]
Influenza A	M2e protein [23]
HIV	gp120 [24,26,27,28,29,30,32] gp41 [29,30] Oligomannose patch on Envs [33]
SARS-CoV-2	S protein [35]
**Other**	
Cardiac fibrosis	Fibroblast activation protein [39]

## Data Availability

Data sharing not applicable.

## Abbreviations

ACT	Adoptive Cell Transfer
ADs	Allergic Diseases
AIDs	Autoimmune Diseases
APC	Antigen-Presenting Cell
BAR	B cell Antibody-targeting Receptor
BCMA	B Cell Maturation Antigen
BCR	B Cell Receptor
BNAbs	Broadly Neutralizing Antibodies
CAAR	Chimeric Autoantibody Receptor
CAR	Chimeric Antigen Receptor
cART	combination Antiretroviral Therapy
CEA	Carcinoembryonic Antigen
CNS	Central Nervous System
CRD	Carbohydrate Recognition Domain
CRS	Cytokine Release Syndrome
CSS	Cytokine Storm Syndrome
CTLA-4	Cytotoxic T-lymphocyte antigen 4
FAP	Fibroblast Activation Protein
gB	Glycoprotein B
GvHD	graft-versus-host disease
HA	Hemophilia A
HBV	Hepatitis B Virus
HCMV	Human Cytomegalovirus
HCV	Hepatitis C Virus
HCV/E2	HCV E2 glycoprotein
HIV-1	Human Immunodeficiency Virus type 1
IFI	Invasive Fungal Infection
IFNγ	Interferon gamma
MHC	major histocompatibility complex
MOG	Myelin Oligodendrocyte Glycoprotein
MS	Multiple Sclerosis
NK	Natural Killer
NMOSD	Neuromyelitis Optica Spectrum Disorder
NOD	Non-obese diabetic
PID	Primary Immune Deficiency
PV	Pemphigus Vulgaris
SARS-CoV-2	Severe Acute Respiratory Syndrome Coronavirus 2
scFv	single-chain Variable fragment
SLE	Systemic Lupus Erythematosus
T1D	Type 1 Diabetes
TCR	T cell receptor
TILs	Tumor-Infiltrating Lymphocytes
TNFα	Tumor Necrosis Factor alpha
Treg	T regulatory lymphocyte
